# Supplementary material for: The Impact of the COVID-19 Pandemic on Immunization Campaigns and Programs: A Systematic Review
Source: Int J Environ Res Public Health. 2021 Jan 22;18(3):988. doi: 10.3390/ijerph18030988 (PMC7908591; doi:10.3390/ijerph18030988)
Supplement: Supplementary file 1 [file ijerph-18-00988-s001.pdf]

## Supplementary tables

**Table S1.** PRISMA Checklist.

| | | | |
|---|---|---|---|
| **TITLE** | | | |
| Title | 1 | Identify the report as a systematic review, meta-analysis, or both. | 1 |
| **ABSTRACT** | | | |
| Structured summary | 2 | Provide a structured summary including, as applicable: background; objectives; data sources; study eligibility criteria, participants, and interventions; study appraisal and synthesis methods; results; limitations; conclusions and implications of key findings; systematic review registration number. | 1 |
| **INTRODUCTION** | | | |
| Rationale | 3 | Describe the rationale for the review in the context of what is already known. | 2 |
| Objectives | 4 | Provide an explicit statement of questions being addressed with reference to participants, interventions, comparisons, outcomes, and study design (PICOS). | 3 |
| **METHODS** | | | |
| Protocol and registration | 5 | Indicate if a review protocol exists, if and where it can be accessed (e.g., Web address), and, if available, provide registration information including registration number. | 3 |
| Eligibility criteria | 6 | Specify study characteristics (e.g., PICOS, length of follow-up) and report characteristics (e.g., years considered, language, publication status) used as criteria for eligibility, giving rationale. | 3 |
| Information sources | 7 | Describe all information sources (e.g., databases with dates of coverage, contact with study authors to identify additional studies) in the search and date last searched. | 3 |
| Search | 8 | Present full electronic search strategy for at least one database, including any limits used, such that it could be repeated. | Supplementary fie 2 |

| | | | |
|---|---|---|---|
| Study selection | 9 | State the process for selecting studies (i.e., screening, eligibility, included in systematic review, and, if applicable, included in the meta-analysis). | 3 |
| Data collection process | 10 | Describe method of data extraction from reports (e.g., piloted forms, independently, in duplicate) and any processes for obtaining and confirming data from investigators. | 3 |
| Data items | 11 | List and define all variables for which data were sought (e.g., PICOS, funding sources) and any assumptions and simplifications made. | 3 |
| Risk of bias in individual studies | 12 | Describe methods used for assessing risk of bias of individual studies (including specification of whether this was done at the study or outcome level), and how this information is to be used in any data synthesis. | 4 |
| Summary measures | 13 | State the principal summary measures (e.g., risk ratio, difference in means). | NA |
| Synthesis of results | 14 | Describe the methods of handling data and combining results of studies, if done, including measures of consistency (e.g., $I^2$) for each meta-analysis. | NA |
| Risk of bias across studies | 15 | Specify any assessment of risk of bias that may affect the cumulative evidence (e.g., publication bias, selective reporting within studies). | NA |
| Additional analyses | 16 | Describe methods of additional analyses (e.g., sensitivity or subgroup analyses, meta-regression), if done, indicating which were pre-specified. | NA |
| **RESULTS** | | | |
| Study selection | 17 | Give numbers of studies screened, assessed for eligibility, and included in the review, with reasons for exclusions at each stage, ideally with a flow diagram. | 4 |
| Study characteristics | 18 | For each study, present characteristics for which data were extracted (e.g., study size, PICOS, follow-up period) and provide the citations. | 4 |
| Risk of bias within studies | 19 | Present data on risk of bias of each study and, if available, any outcome level assessment (see item 12). | 4 |
| Results of individual studies | 20 | For all outcomes considered (benefits or harms), present, for each study: (a) simple summary data for each intervention group (b) effect estimates and confidence intervals, ideally with a forest plot. | 4-7 |

| | | | |
|---|---|---|---|
| Synthesis of results | 21 | Present results of each meta-analysis done, including confidence intervals and measures of consistency. | NA |
| Risk of bias across studies | 22 | Present results of any assessment of risk of bias across studies (see Item 15). | 18-19 |
| Additional analysis | 23 | Give results of additional analyses, if done (e.g., sensitivity or subgroup analyses, meta-regression [see Item 16]). | NA |
| **DISCUSSION** | | | |
| Summary of evidence | 24 | Summarize the main findings including the strength of evidence for each main outcome; consider their relevance to key groups (e.g., healthcare providers, users, and policy makers). | 7-8 |
| Limitations | 25 | Discuss limitations at study and outcome level (e.g., risk of bias), and at review-level (e.g., incomplete retrieval of identified research, reporting bias). | 9 |
| Conclusions | 26 | Provide a general interpretation of the results in the context of other evidence, and implications for future research. | 9 |
| **FUNDING** | | | |
| Funding | 27 | Describe sources of funding for the systematic review and other support (e.g., supply of data); role of funders for the systematic review. | 10 |

**File S2.** Search strategy.Number of results on pubmed: 3579 titles.

(coronavirus 2019[tw] OR nouveau coronavirus[tw] OR corona virus 2019[tw] OR coronavirus disease 2019[tw] OR corona virus disease 2019[tw] OR coronavirus pandemic[tw] OR corona virus pandemic[tw] OR coronavirus crisis[tw] OR corona virus crisis[tw] OR coronavirus outbreak[tw] OR corona virus outbreak[tw] OR "coronavirus disease -19"[tw] OR "corona virus disease -19"[tw] OR coronavirus epidemic[tw] OR corona virus epidemic[tw] OR coronavirus disease 2019 virus[tw] OR novel coronavirus[tw] OR novel corona virus[tw] OR novel coronaviruses[tw] OR "2019 novel coronavirus"[tw] OR new coronavirus[tw] OR new corona virus[tw] OR new coronaviruses[tw] OR covid-19[tw] OR covid-19 pandemic[tw] OR covid-19 virus[tw] OR covid19 virus[tw] OR covid-19 crisis[tw] OR covid-19 outbreak[tw] OR covid-19 infection*[tw] OR covid-19 virus infection*[tw] OR severe acute respiratory syndrome coronavirus 2[tw] OR SARS-CoV-2[tw] OR SARS coronavirus 2[tw] OR SARS-CoV-2 infection*[tw] OR nCoV OR "2019 - nCoV"[tw] OR "2019 - nCoV disease"[tw] OR "2019 - nCoV infection"[tw]) AND

(Immunization[Mesh] OR immuni*[tw] OR Vaccines[Mesh] OR Vaccination[Mesh] OR vaccin*[tw] OR "Papillomavirus Vaccines"[Mesh] OR Papillomavirus[tw] OR “Papillomaviridae”[Mesh] OR HPV[tw] OR "wart virus"[tw] OR “Warts”[Mesh] OR "wart virus"[tw] OR "Condylomata Acuminata"[Mesh] OR "Measles-Mumps-Rubella Vaccine"[Mesh] OR MMR[tw] OR “Measles”[Mesh] OR "Measles virus"[Mesh] OR "Measles Vaccine"[Mesh] OR mumps*[tw] OR Mumps[Mesh] OR "Mumps virus"[Mesh] OR "Mumps Vaccine"[Mesh] OR Rubella[Mesh] OR "Rubella virus"[Mesh] OR "Rubella Vaccine"[Mesh] OR varicella[tw] OR “Chickenpox”[Mesh] OR "Chickenpox Vaccine"[Mesh] OR zoster[tw] OR "Herpes Zoster"[Mesh] OR "Herpes Zoster Vaccine"[Mesh] OR "Diphtheria-Tetanus Vaccine"[Mesh] OR tetanus[tw] OR "Diphtheria-Tetanus-Pertussis Vaccine"[Mesh] OR Tetanus[Mesh] OR "Tetanus Toxoid"[Mesh] OR "Diphtheria-Tetanus-acellular Pertussis Vaccines"[Mesh] OR "Whooping Cough"[Mesh] OR Diphtheria[Mesh] OR vzv[tw] OR "Bordetella pertussis"[Mesh] OR "Haemophilus Vaccines"[Mesh] OR pertusis[tw] OR tdap[tw] OR "Meningococcal Infections"[Mesh] OR meningococcal[tw] OR "Meningitis, Meningococcal"[Mesh] OR "Meningococcal Vaccines"[Mesh] OR mcv[tw] OR "Meningitis, Pneumococcal"[Mesh] OR "Pneumococcal Infections"[Mesh] OR "Pneumococcal Vaccines"[Mesh] OR "Hepatitis A"[Mesh] OR "Hepatitis A Vaccines"[Mesh] OR "Hepatitis B"[Mesh] OR "Hepatitis B Vaccines"[Mesh] OR "Hepatitis C"[Mesh] OR polio[tw] OR Poliomyelitis[Mesh] OR "Poliovirus Vaccines"[Mesh] OR "Rotavirus Infections"[Mesh] OR Rotavirus[Mesh] OR "Rotavirus Vaccines"[Mesh] OR "typhoid vaccine"[tw] OR "Typhoid-Paratyphoid Vaccines"[Mesh] OR typhoid[tw] OR "Typhoid Fever"[Mesh] OR influenza[tw] OR "Influenza, Human"[Mesh] OR "Influenza Vaccines" OR "BCG Vaccine" OR " Bacterial Vaccines"[Mesh] OR "Vaccine-Preventable Diseases"[Mesh] OR "Immunization Programs"[Mesh] OR immunization campaign*[tw] OR immunization promotion[tw] OR immunization awareness[tw] OR routine immunization*[tw] OR routine vaccin*[tw] OR catch-up immunization*[tw] OR "Immunization Schedule"[Mesh] OR vaccination schedule[tw] OR "Vaccination Coverage"[Mesh] OR mop-up campaign*[tw] OR Global Polio Eradication Initiative OR Polio eradication initiative*[tw] OR "Mass Vaccination"[Mesh] OR expanded program* on immunization[tw] OR EPI[tw])
